# Letter regarding “Associations of non-HDL-C and triglyceride/HDL-C ratio with coronary plaque burden and plaque characteristics in young adults”

**DOI:** 10.17305/bjbms.2022.7782

**Published:** 2023-01-06

**Authors:** Gulali Aktas

**Affiliations:** 1 Faculty of Medicine, Abant Izzet Baysal University, Bolu, Turkey

Dear Editor:

I read with great interest the article by Akin et al., reporting an association between coronary plaque and serum lipid parameters [1]. The authors examined serum lipid levels in patients undergoing computed tomography of the coronary arteries and reported that LDL cholesterol, total cholesterol, non-HDL cholesterol, triglycerides, and the ratio of triglycerides to HDL cholesterol were significantly higher in patients with coronary artery disease, and HDL cholesterol was significantly lower than in patients without coronary atherosclerosis. The results presented in the above study are consistent with data from the literature. Increased total and LDL cholesterol levels were found in patients with adequate coronary collateral development compared with patients with inadequate coronary collateral development [2].

However, neither triglyceride nor HDL cholesterol levels differed significantly between subjects with and without adequate coronary collateral development [2]. Calculation of the triglyceride/HDL ratio has been widely used in recent research because it is an easily determined and inexpensive marker. Elevated levels of the triglyceride/HDL cholesterol ratio are considered a risk factor for coronary atherosclerosis [3]. In addition, the triglyceride/HDL-cholesterol ratio has been proposed as a new index of worsened metabolism and increased inflammatory burden. In a recent study, diabetic patients with chronic complications were found to have an increased triglyceride/HDL-cholesterol ratio compared with individuals without complications [4]. In addition, individuals with intracranial atherosclerosis have a higher triglyceride/HDL-cholesterol ratio than patients without intracranial atherosclerosis [5]. High triglyceride/HDL-cholesterol ratios in diabetics, in patients with intracranial atherosclerosis, and in individuals with coronary artery disease are not surprising, because type 2 diabetes mellitus is a risk factor for the development of atherosclerosis in the coronary arteries as well as in other arterial circulations. There is also controversial work in the literature. In a Mayo Clinic study, the authors found no significant difference in the ratio between triglycerides and HDL cholesterol in subjects with severe cardiac events compared with subjects without severe cardiac events.

In conclusion, because of its simple and inexpensive determination, the triglyceride/HDL ratio is a useful tool for evaluating poor metabolic outcomes in a variety of conditions, from type 2 diabetes mellitus to atherosclerotic disease.

**Conflicts of interest:** Author declares no conflicts of interest.

**Funding:** Author received no specific funding for this work
